# *TOP1MT* deficiency promotes GC invasion and migration via the enhancements of LDHA expression and aerobic glycolysis

**DOI:** 10.1530/ERC-17-0058

**Published:** 2017-09-05

**Authors:** Hongqiang Wang, Rui Zhou, Li Sun, Jianling Xia, Xuchun Yang, Changqie Pan, Na Huang, Min Shi, Jianping Bin, Yulin Liao, Wangjun Liao

**Affiliations:** 1 Department of OncologyNanfang Hospital, Southern Medical University, Guangzhou, China; 2 Department of OncologyZhoushan Hospital, Zhoushan, China; 3 Department of CardiologyNanfang Hospital, Southern Medical University, Guangzhou, China

**Keywords:** glycolysis, gastric cancer, lactate dehydrogenase A, mitochondrial topoisomerase I

## Abstract

Aerobic glycolysis plays an important role in cancer progression. New target genes regulating cancer aerobic glycolysis must be explored to improve patient prognosis. Mitochondrial topoisomerase I (*TOP1MT*) deficiency suppresses glucose oxidative metabolism but enhances glycolysis in normal cells. Here, we examined the role of *TOP1MT* in gastric cancer (GC) and attempted to determine the underlying mechanism. Using *in vitro* and *in vivo* experiments and analyzing the clinicopathological characteristics of patients with GC, we found that *TOP1MT* expression was lower in GC samples than in adjacent nonmalignant tissues. *TOP1MT* knockdown significantly promoted GC migration and invasion *in vitro* and *in vivo*. Importantly, *TOP1MT* silencing increased glucose consumption, lactate production, glucose transporter 1 expression and the epithelial-mesenchymal transition (EMT) in GC. Additionally, regulation of glucose metabolism induced by *TOP1MT* was significantly associated with lactate dehydrogenase A (LDHA) expression. A retrospective analysis of clinical data from 295 patients with GC demonstrated that low *TOP1MT* expression was associated with lymph node metastasis, recurrence and high mortality rates. *TOP1MT* deficiency enhanced glucose aerobic glycolysis by stimulating LDHA to promote GC progression.

## Introduction

Metabolic disturbance is a universal property of cancer cells and one of the most important causes of tumorigenesis and cancer progression (Hsu & Sabatini 2008, Kroemer & Pouyssegur 2008, Lu *et al*. 2015). The main metabolism-related characteristic of most cancer cells is a metabolic switch from the mitochondrial tricarboxylic acid (TCA) cycle to aerobic glycolysis. Even under aerobic conditions, glucose is taken up and transformed to lactate via the glycolytic pathway in cancer cells, a phenomenon known as the Warburg effect (Warburg 1956). This metabolic switch produces an acidic microenvironment for the tumor and promotes the epithelial-mesenchymal transition (EMT) and metastasis, leading to poor prognosis (Vander Heiden *et al*. 2009, Hirschhaeuser *et al*. 2011, Chen 2012, Lu *et al*. 2015). Studies of the Warburg effect in tumors have been a major focus of tumor metabolism research. Therefore, analysis of the Warburg effect and exploring the underlying target genes may provide clues for therapeutic strategies in clinical oncology.

Mitochondrial topoisomerase I (*TOP1MT*) encodes a crucial mitochondrial DNA topoisomerase that plays an important role in relieving mitochondrial DNA tension and supercoiling produced during replication and transcription (Zhang *et al*. 2001). *TOP1MT* deficiency influences mitochondrial DNA expression, which decreases the expression of genes in the oxidative phosphorylation (OXPHOS) system involved in both OXPHOS and electron transport in normal cells (DiMauro & Schon 1998, Zhang *et al*. 2001). Additionally, defective *TOP1MT* gene expression may predispose individuals to various diseases related to mitochondrial disorders, e.g., diabetes, Alzheimer’s disease and Parkinson’s syndrome. Interestingly, *TOP1MT* is involved in mitochondrial OXPHOS not only in normal cells but also in cancer cells (Goto *et al*. 2006). However, the role of *TOP1MT* in cancer cells has not been reported.

In normal cells, the mechanism through which *TOP1MT* affects metabolism involves enhancement of OXPHOS (Douarre *et al*. 2012). *TOP1MT* deficiency leads to mitochondrial respiratory chain defects, which inhibit the mitochondrial TCA cycle and OXPHOS. However, through alternative pathways, cells with *TOP1MT* deficiency enhance glycolysis for energy production. In this process, the TCA cycle and OXPHOS decrease, glucose consumption and lactate production increase and glycolytic enzymes are concomitantly activated. For example, lactate dehydrogenase A (LDHA) is a glycolytic enzyme that catalyzes the formation of lactate from pyruvate. Even in cancer cells, LDHA plays important role in controlling the speed of glycolysis and maintaining the continuity of aerobic glycolysis (Fantin *et al*. 2006, Ganapathy *et al*. 2009). Studies have demonstrated that aerobic glycolysis promotes cancer cell progression (Chen 2012, Lin *et al*. 2015, Lu *et al*. 2015), and mitochondrial OXPHOS suppresses tumor metastasis (Lu *et al*. 2015); thus, metabolic therapies are potential therapeutic strategies for prevention or intervention of cancer metastasis (Kolev *et al*. 2008, Le *et al*. 2010, Fiume *et al*. 2014, Augoff *et al*. 2015). In fact, studies on tumor metabolism have demonstrated that knowledge of tumor glycolysis may facilitate the discovery and development of therapeutic strategies for clinical oncology (Giatromanolaki *et al*. 2006, Koukourakis *et al*. 2006, 2009, Chen 2012). Moreover, analysis of *TOP1MT* may provide clues for therapeutic strategies by regulating the Warburg effect in tumors.

Therefore, in this study, we sought to determine the role of *TOP1MT* in invasion and migration and elucidate the regulatory metabolic mechanisms through which *TOP1MT* is involved in GC. We discovered that *TOP1MT* deficiency promoted GC cell invasion and migration; increased glucose consumption, lactate production and LDHA activity *in vitro* and *in vivo* and promoted tumor metastasis in patients with GC. This study is the first report demonstrating the roles of *TOP1MT* in GC and the mechanisms through which *TOP1MT* regulates the Warburg effect to promote GC progression.

## Materials and methods

### Patients and tissue samples

All patients enrolled in this study provided informed consent, and the Nanfang Hospital Ethics Review Board approved our research. Fresh tissue specimens were obtained from 12 patients who underwent radical surgery for GC between May and June 2015. Paraffin-embedded tissue specimens for immunohistochemistry were obtained from 295 patients with GC who had not undergone any neoadjuvant chemotherapy before surgery in our hospital between 2006 and 2011.

### Real-time quantitative reverse transcription polymerase chain reaction (RT-PCR)

Total RNAs were acquired from GC specimens and cell lines using TRIzol reagent and reverse transcribed with M-MLV reverse transcriptase. Real-time PCR was conducted using a SYBR Green I Master kit on a LightCycler 480 system. β-Actin expression was determined as an internal control. The primer sequences for real-time RT-PCR are shown in Supplementary Table 1 (see section on supplementary data given at the end of this article).

### Western blotting

Cells were lysed in cell lysis buffer. Equal amounts of proteins from cell extracts were resolved by sodium dodecyl sulfate polyacrylamide gel electrophoresis on 10% gels and transferred to polyvinylidene difluoride membranes (Millipore). The blots were blocked in a solution of 10% skimmed milk and incubated with primary antibodies overnight at 4°C. Subsequently, the blots were washed and incubated with secondary antibodies for 1 h. Antibody-bound protein bands were detected using an Odyssey Infrared Imaging System (LI-COR Biosciences, Lincoln, NE, USA).

### Immunohistochemistry

Immunohistochemical staining was performed according to a published standard protocol (Wang *et al*. 2013, Lin *et al*. 2015, Xia *et al*. 2016). The staining intensity was scored as 0 (no staining), 1 (weak staining), 2 (medium staining) or 3 (strong staining). Staining extent was scored based on the percentage of positive cells as 0 (0%), 1 (1%–25%), 2 (26%–50%), 3 (51%–75%) or 4 (76%–100%). The final staining scores (0–12) for TOP1MT and LDHA expression were obtained by multiplying the intensity and the extent scores. Tumor tissues with final staining scores of less than 6 and greater than or equal to 6 were regarded as having low and high TOP1MT expression, respectively.

### Methyl thiazolyl tetrazolium (MTT) assay

Cell proliferation was measured using MTT assays. Briefly, BGC-823 or SGC-7901 cells were seeded into 96-well plates at a density of 4 × 10^3^ cells/well. On the indicated days after plating, 200 μL MTT solution (5 mg/mL) was added to each well at 0, 24, 48, 72 and 96 h. The samples were then incubated for 4 h at 37°C. Subsequently, the medium was replaced with 150 μL dimethyl sulfoxide for an additional 10-min incubation. The absorbance was then determined at 570 nm with a microplate reader (Molecular Devices).

### Colony formation assay

A total of 200 GC cells were seeded onto 6-well culture dishes. After 14 days of culture, the cells were fixed with 4% paraformaldehyde and stained with Giemsa solution. Colonies containing at least 50 cells in each well were counted using the following formula: plate clone formation efficiency = (number of colonies/number of cells inoculated) × 100%.

### Wound healing assay

Cells were plated in 6-well plates at a density of 5 × 10^4^ cells/well. After cells reached confluence, they were scratched using a sterile plastic tip. Wound closure was visualized at 0 h and 24 h using an inverted microscope.

### Transwell migration assay

Cells (1 × 10^5^/mL, 200 µL) in RPMI-1640 (Invitrogen, Life Technologies) without serum were plated in the upper chambers of wells containing 8-µm pore size polycarbonate filters (Costar, Bodenheim, Germany). The lower chamber was filled with 600 µL RPMI-1640 containing 10% fetal bovine serum (FBS). Cells were incubated for 24 h at 37°C. The upper chambers were washed twice. Cells invading through chambers and adhering to the lower surface were fixed with 4% paraformaldehyde for 15 min and stained with hematoxylin for 20 min. The invading cells on the lower surface of the chamber were photographed and counted under a microscope in five randomly selected fields.

### Boyden chamber invasion assays

Cell invasion assays were similar to transwell migration assays, except that the polycarbonate filters of the upper chamber were coated with Matrigel (BD Biosciences).

### Knockdown of target genes by small interfering RNAs (siRNAs)

Transfection of BGC-823 and SGC-7901 cells with siRNAs was performed using Lipofectamine 2000. For transient transfection, cells were transfected with siRNAs for 6 h and then cultured in RPMI-1640 with 10% FBS for 48 h before subsequent functional assays. The sequences of siRNAs for *TOP1MT* and *LDHA* are listed in Supplementary Table 2. Cells treated with the transfection reagents alone were included as negative controls.

### Glucose consumption, lactate production, pH and LDHA activity assays

As in our previous study (Lin *et al*. 2015), 1 × 10^5^ cells were seeded in each well of 6-well plates and incubated under normoxic conditions for 24 h at 37°C. The culture medium of cells was collected for lactate, glucose and pH assays. Lactate and glucose concentrations were measured using enzymatic methods (Randox, Crumlin, Antrim, UK). pH values were determined using a pH meter. Cytoplasmic LDHA activity was measured with assay kits (Comin, Suzhou, China).

### ATP determination

Cellular ATP generation was determined using an ATP determination kit based on firefly luciferase (Beyotime, Haimen, China).

### Cell culture and stable cell lines

Cell culture and stable cell line establishment were performed as previously described (Wang *et al*. 2013). The human gastric epithelial cell line GES-1 and GC cell lines BGC-823, AGS, MKN-7, SGC-7901, MKN-28, MKN-45 and MGC-803 were purchased from Foleibao Biotechnology Development Company (Shanghai, China). Molecular authentication of each cell line was performed by short tandem repeat analysis, and passage numbers used for the experiments are provided (Supplementary Fig. 1). Cells were cultured in RPMI-1640 with 10% FBS at 37°C in 5% CO_2_. Sodium oxamate, an LDHA inhibitor, was obtained from Sigma–Aldrich. In the *in vivo* assay, cells exhibiting stable *TOP1MT* knockdown were established by transfection of BGC-823 and SGC-7901 cells with short hairpin RNA targeting *TOP1MT* (*shTOP1MT*) (Wang *et al*. 2013).

### Xenograft model

The influence of *TOP1MT* on tumor metastasis was further demonstrated *in vivo*. All null nude mice were treated according to ethical guidelines for the care and use of experimental animals. *shTOP1MT* and scramble control (5 × 10^6^) GC cells (BGC-823 and SGC-7901 cell lines) were subcutaneously injected into the flanks of 4-week-old female null nude mice (*n* = 40, 10 mice per cell line). Tumor sizes were evaluated every 4 days with a digital caliper. Tumor volumes were obtained as follows: volume = width × length × (width + length)/2. On day 28 after inoculation, the mice were killed, and tumors were harvested and weighted. To test the influence of *TOP1MT* on tumor metastasis *in vivo*, null nude mice were subjected to caudal vein injection (2 × 10^6^ cells/mouse, *n* = 48, 12 mice per cell line) of BGC-823 and SGC-7901 cells with *shTOP1MT* or scramble control shRNA. The molecular changes in cells with or without *TOP1MT* silencing were confirmed by Western blotting, and LDHA expression was checked by immunohistochemistry. On day 40 after caudal vein injection, the mice were killed and dissected. Pulmonary metastasis nodules were counted and visualized by hematoxylin and eosin staining.

### Statistical analysis

Unless otherwise indicated, all assays were repeated at least three times, and representative results were reported as means ± standard deviations (s.d.). Statistical significance was determined by Student’s *t*-test or one-way analysis of variance (ANOVA) with Tukey’s multiple comparison tests. Correlations between two continuous variables were examined by Pearson’s test. Survival rates were analyzed by the Kaplan–Meier method and examined by log-rank tests. Differences with *P* values of less than 0.05 were considered statistically significant. The SPSS13.0 software program was used for analyzing all data.

## Results

### Decreased expression of *TOP1MT* in GC

**Figure 1:**
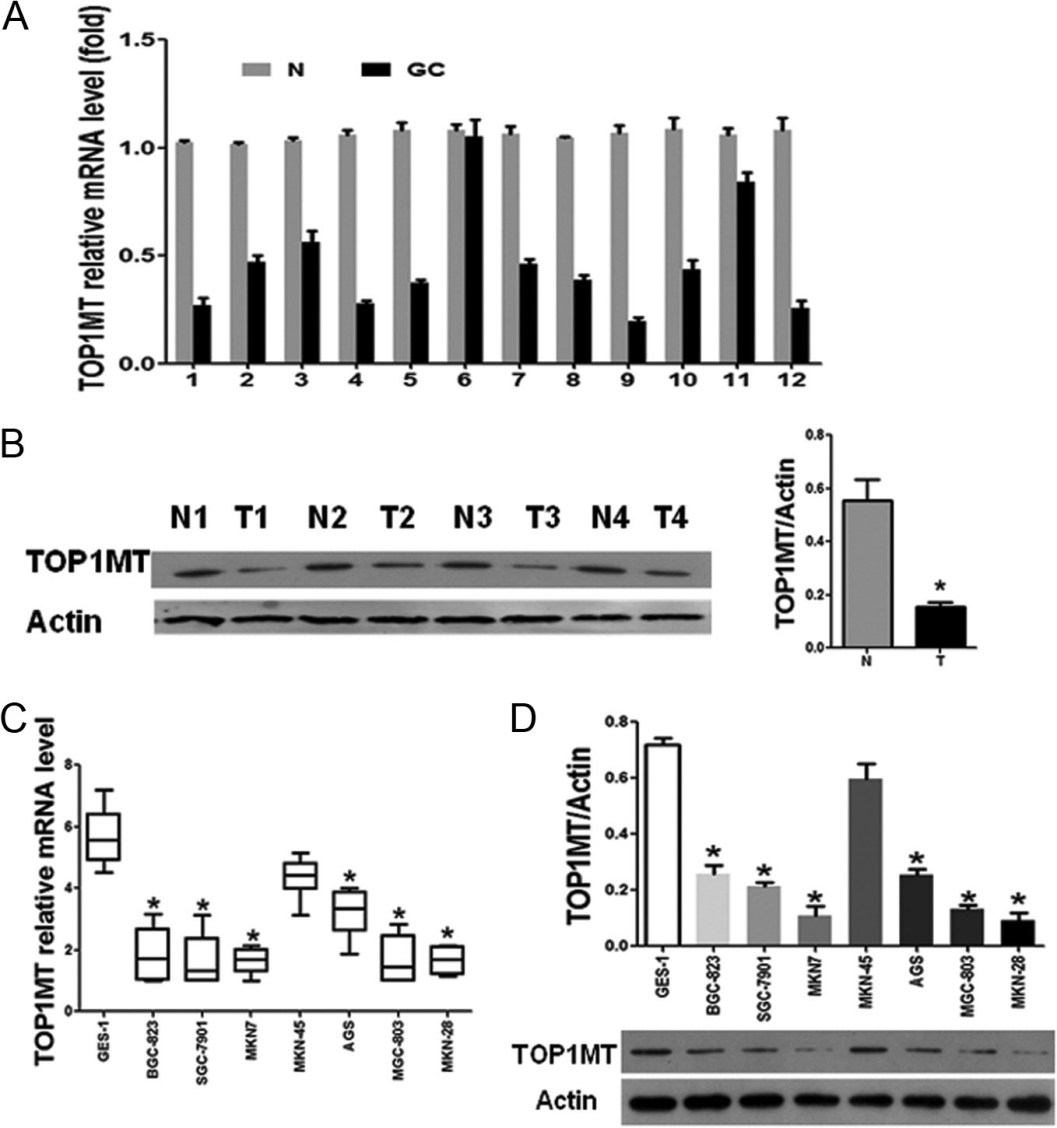
*TOP1MT* expression was downregulated in gastric cancer tissues and cell lines. (A and B) *TOP1MT* expression levels in gastric cancer (GC) tissues from patients and normal adjacent tissues (N) were analyzed by RT-PCR (A) and Western blotting (B). (C and D) *TOP1MT* expression levels in GC cell lines and GES-1 normal gastric epithelial cells were analyzed by RT-PCR (C) and Western blotting (D). *P < 0.05 in comparisons of GC tissues (T) or GC cell lines with normal adjacent tissues (N) groups or GES-1 normal gastric epithelial cells.

We first detected the RNA expression of *TOP1MT* in tissue by RT-PCR and found that *TOP1MT* mRNA levels were lower (*P* < 0.05) in GC tissues than in adjacent normal tissues (Fig. 1A). Western blotting results also showed that TOP1MT expression was significantly lower in GC tissue samples (Fig. 1B).

We further examined *TOP1MT* expression in seven human GC cell lines (BGC-823, AGS, MKN-7, SGC-7901, MKN-28, MKN-45 and MGC-803) and a normal gastric epithelial cell line (GES-1). We also found that *TOP1MT* expression was lower at both mRNA (Fig. 1C) and protein levels (Fig. 1D) in GC cell lines compared with that in GES-1 cells.

### Silencing of *TOP1MT* enhanced the migration and invasion of GC *in vitro* and *in vivo*

Our initial results demonstrated that *TOP1MT* expression was low in both GC tissue samples and cell lines. However, it was still unclear whether this low *TOP1MT* expression affected the functions of GC cells. To explore the effects of low *TOP1MT* expression on GC cells, we performed functional experiments in BGC-823 and SGC-7901 cells. Cells were transiently transfected with siRNA targeting *TOP1MT*. Both *siTOP1MT1* and *siTOP1MT2* significantly inhibited *TOP1MT* mRNA (Supplementary Fig. 2A) and protein expression (Supplementary Fig. 2B and C). *siTOP1MT1* was selected for the following functional experiments because of its higher inhibitory effect. To demonstrate that the *shTOP1MT* constructs specifically targeted the mitochondrial isoform of TOP1, mRNA and protein levels of TOP1, TOP2α, TOP2β, TOP3α and TOP3β were tested by RT- PCR and Western blotting. *TOP1MT* knockdown did not affect the mRNA or protein levels of these targets in GC cells (Supplementary Fig. 3C, D and E).

**Figure 2:**
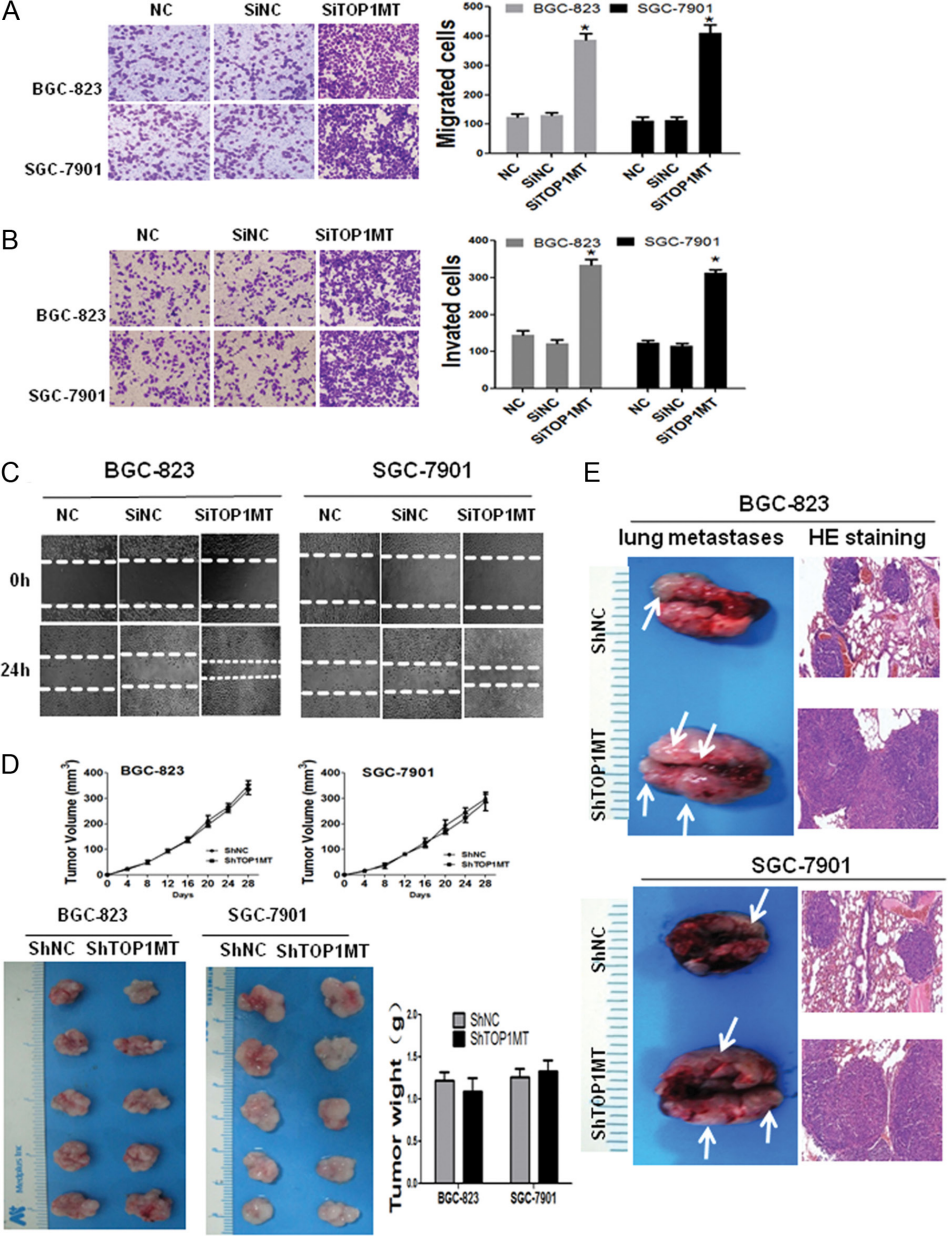
*TOP1MT* silencing enhanced the invasion and migration of BGC-823 and SGC-7901 cells *in vitro* and *in vivo*. (A, B and C) Migration and invasion of GC cells infected with *TOP1MT* siRNA (*SiTOP1MT*) or negative control siRNA (SiNC) or negative control (NC) were assessed by transwell migration assays (A), Boyden chamber invasion transwell assays (B), and wound closure assays (C). (D) BGC-823/scramble, BGC-823/*shTOP1MT*, SGC-7901/scramble, and SGC-7901/*shTOP1MT* were subcutaneously injected into the flanks of female athymic mice. The volumes (D, upper) of the xenotransplanted tumors were calculated every 4 days. Representative images of subcutaneous xenotransplanted tumors (D, lower left) and tumor weights (D, lower right) were measured when the mice were killed on day 28 post implantation. (E) Gross lung metastases (E, left) and lung metastases stained with hematoxylin and eosin (HE staining) (E, right) for BGC-823 and SGC-7901 cells. *P < 0.05 in comparisons of the *SiTOP1MT*-transfected group with the SiNC or NC group. A full colour version of this figure is available at http://dx.doi.org/10.1530/ERC-17-0058.

Methyl thiazolyl tetrazolium (MTT) assays and colony formation assays showed that *TOP1MT* silencing failed to influence the proliferation of GC cells (Supplementary Fig. 3A and B). However, in transwell assays, the numbers of migrating (Fig. 2A) and invading cells (Fig. 2B) were significantly higher for siRNA-transfected BGC-823 and SGC-7901 cells. In addition, wound-closure migration assays also showed that *TOP1MT* silencing significantly accelerated wound recovery (Fig. 2C).

Furthermore, to determine the effects of low *TOP1MT* expression *in vivo*, *TOP1MT* silenced (*shTOP1MT*) BGC-823 and SGC-7901 cells and their corresponding negative controls, BGC-823/scramble and SGC-7901/scramble were established. BGC-823/*shTOP1MT*, BGC-823/scramble, SGC-7901/*shTOP1MT* and SGC-7901/scramble cells were subcutaneously injected into 40 athymic female mice. The results showed that *TOP1MT* knockdown did not influence tumor growth (Fig. 2D). We assessed the metastasis of GC cells by injecting BGC-823/*shTOP1MT*, BGC-823/scramble, SGC-7901/*shTOP1MT* and SGC-7901/scramble cells into the caudal vein of 48 athymic female mice. Again, *TOP1MT* knockdown increased pulmonary metastasis in both cell lines, as demonstrated by anatomical pathology and histopathology (Fig. 2E). These results clearly indicated that *TOP1MT* silencing promoted the invasion and metastasis of GC cells.

### Silencing of *TOP1MT* upregulated glycolysis in GC cells

**Figure 3:**
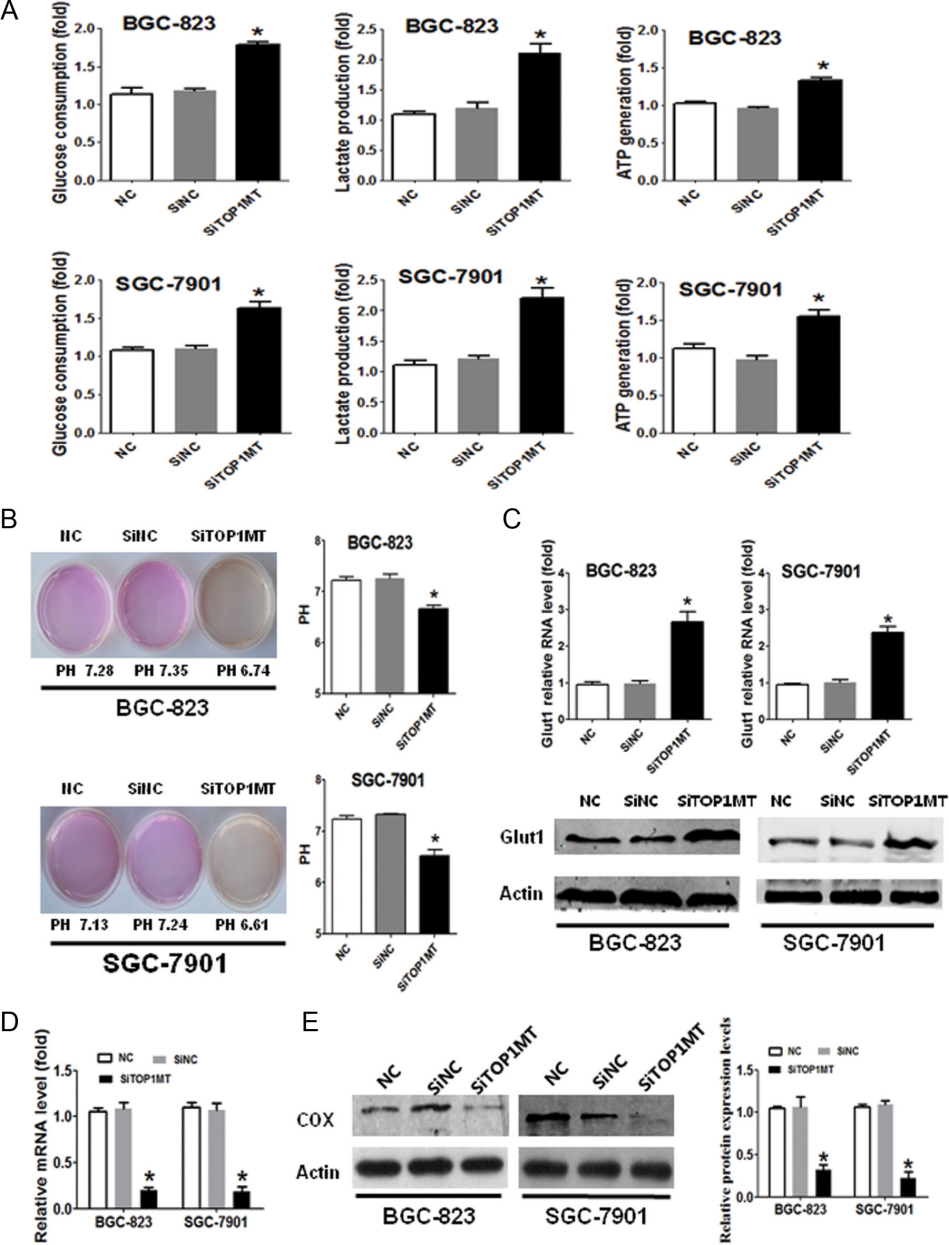
*TOP1MT* silencing upregulated glucose consumption, lactate production and glucose transporter 1 in GC cells. (A) GC cells of NC, SiNC and *SiTOP1MT* groups were incubated for 48 h. Glucose uptake, lactate production and ATP generation in different groups of BGC-823 (A, upper) and SGC-7901 (A, lower) cells were measured using enzymatic methods or assay kits. (B) Representative experiments showing pH determination after 48 h of culture in NC, SiNC and *SiTOP1MT* groups. (C) Representative experiments showing glucose transporter 1 (Glut1) mRNA and protein expression, as examined by RT-PCR and Western blotting in NC, SiNC and *SiTOP1MT* groups. (D and E) Representative experiments showing cytochrome C oxidase (COX) mRNA (D) and protein expression (E), examined by RT-PCR and Western blotting in NC, SiNC and *SiTOP1MT* groups. *P < 0.05 in comparisons of the *SiTOP1MT*-transfected group with the SiNC or NC group. A full colour version of this figure is available at http://dx.doi.org/10.1530/ERC-17-0058.

Given that *TOP1MT* deficiency was found to promote GC cell invasion and migration, we further explored the potential mechanisms. In normal cells, *TOP1MT* silencing has been found to enhance cell glycolysis (Douarre *et al*. 2012), and in cancer cells, glycolysis promotes cell metastasis (Chen 2012, Lin *et al*. 2015, Lu *et al*. 2015). Therefore, enhanced glycolysis induced by *TOP1MT* deficiency may be a key causal factor in promoting GC progression. We explored the impact of *TOP1MT* on glycolysis in GC cells. *TOP1MT* silencing significantly increased glucose utilization, lactate production and ATP generation (Fig. 3A) and decreased the pH (Fig. 3B) of the supernatants in BGC-823 and SGC-7901 cells. Increased glucose transporter 1 (Glut1) expression further verified the upregulation of glucose metabolism in cells with *TOP1MT* silencing (Fig. 3C). These results indicated that glycolysis was enhanced in GC cells with *TOP1MT* silencing.

To further explore the effects of *TOP1MT* knockdown on mitochondria in GC cells, we detected cytochrome C oxidase (COX) expression as a marker of OXPHOS activity in mitochondrial respiration (Sotgia *et al*. 2012). Compared with the control, *siTOP1MT* significantly decreased the mRNA and protein expression of COX in GC cells (Fig. 3D and E).

### Silencing of *TOP1MT* induced GC cell progression by targeting *LDHA*

**Figure 4:**
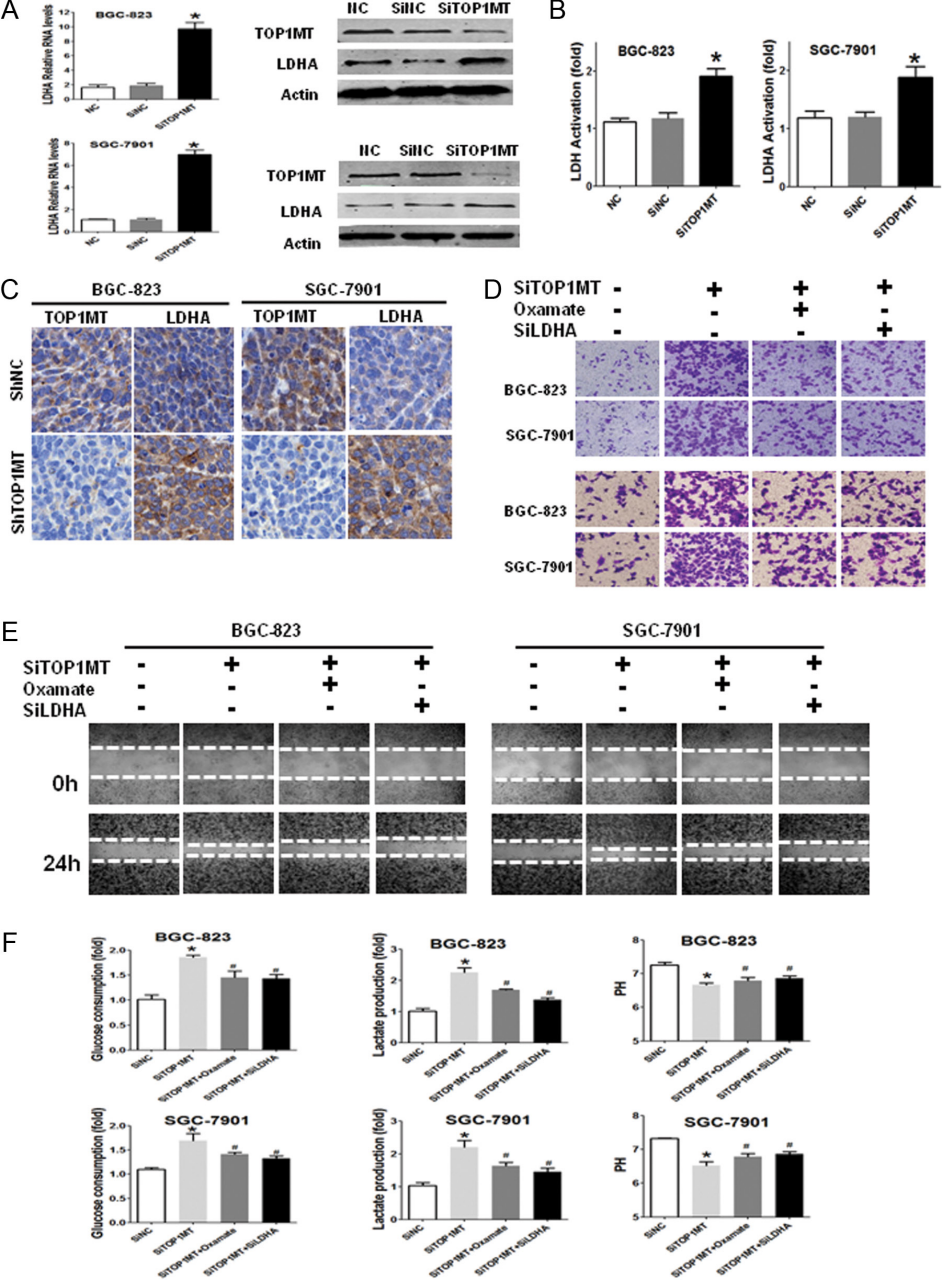
LDHA was required for the upregulation of migration, invasion and glycolysis in GC cells induced by *TOP1MT* silencing. (A) RT-PCR and Western blot analysis for the detection of LDHA expression in BGC-823 and SGC-7901 cells. (B) LDHA activation was examined for BGC-823 and SGC-7901 cells using LDHA activity assays. (C) Representative images of immunohistochemical staining of LDHA expression for BGC-823 and SGC-7901 cells in subcutaneous xenografts. LDHA expression significantly increased in BGC-823 and SGC-7901 cells with *TOP1MT* knockdown. (D, E and F) BGC-823 and SGC-7901 cells transfected with *SiTOP1MT* and/or cultured with LDHA inhibitor (oxamate sodium or *siLDHA*) were subjected to transwell migration assays (D, upper), Boyden chamber invasion transwell assays (D, lower) and wound closure assays (E) to analyze the invasion and migration of GC cells. The results demonstrated LDH inhibition counteracted the migration and invasion induced by *TOP1MT* knockdown. (F) The results showed that LDHA inhibition reversed the enhancement of glucose consumption (F, left), lactate produced (F, middle) in BGC-823 and SGC-7901 cells induced by *TOP1MT* silencing, leading to PH values increase (F, right). *P < 0.05 in comparisons of the *SiTOP1MT*-transfected group with the control group. ^#^ *P* < 0.05 in comparisons of the *SiTOP1MT* with oxamate/*SiLDHA* group with the *SiTOP1MT* group. A full colour version of this figure is available at http://dx.doi.org/10.1530/ERC-17-0058.

To further demonstrate the underlying mechanisms through which *TOP1MT* promoted GC progression, 227 genes related to glycolysis were analyzed by RT-PCR arrays. The results (Supplementary Fig. 4A) showed that *LDHA* mRNA expression was higher than that of other 227 genes associated with glycolysis and oxidative phosphorylation in BGC-823 cells. Both *siLDHA1* and *siLDHA2* significantly inhibited *LDHA* mRNA (Supplementary Fig. 4B) and protein expression (Supplementary Fig. 4C and D). *siLDHA1* was selected and further tests were performed to verify LDHA function in GC cell lines. *LDHA* mRNA and protein expression (Fig. 4A and Supplementary Fig. 4E) and LDHA activation (Fig. 4B) were significantly increased in BGC-823 and SGC-7901 cells with *TOP1MT* knockdown. Immunohistochemical staining results of subcutaneous xenograft tumors showed that *TOP1MT* knockdown enhanced LDHA expression (Fig. 4C) *in vivo*, consistent with the results of the GC cell lines *in vitro*. Taken together, these results demonstrated that LDHA expression and activation were enhanced in GC cells with *TOP1MT* silencing *in vitro* and *in vivo.*

To determine whether GC cell progression was associated with increased LDHA expression, we exposed cells to oxamate, a well-known inhibitor of LDHA, and *siLDHA*. Our results showed that sodium oxamate or *siLDHA* counteracted the increased migration and invasion induced by *TOP1MT* silencing (Fig. 4D and Supplementary Fig. 4F). The results of wound-closure migration assays further verified these results. The inhibition of LDHA by sodium oxamate or *siLDHA* counteracted the increased wound recovery induced by *TOP1MT* silencing (Fig. 4E). We also found that sodium oxamate or *siLDHA* reversed the enhancement of glucose utilization and lactate production and the decrease in pH values of the supernatants in BGC-823 and SGC-7901 cells induced by *TOP1MT* silencing (Fig. 4F).

### LDHA was associated with changes in EMT markers induced by *TOP1MT* silencing

**Figure 5:**
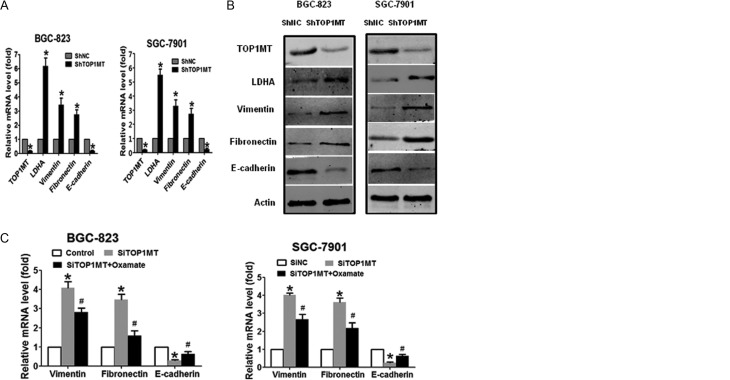
LDHA was associated with changes in EMT markers induced by *TOP1MT* silencing (A) mRNA and (B) protein expression of vimentin, fibronectin, and E-cadherin in BGC-823 and SGC-7901 cells transfected with *shTOP1MT* or the scramble control (ShNC) were analyzed by RT-PCR and Western blotting, respectively. (C and D) BGC-823 and SGC-7901 cells infected with *SiTOP1MT* or inhibited by oxamate sodium were subjected to RT-PCR (C) and Western blotting (D) for detection of vimentin, fibronectin, and E-cadherin mRNA and protein expression, respectively. *P < 0.05 in comparisons of the *ShTOP1MT*-/*SiTOP1MT*-transfected groups with the ShNC or SiNC group. ^#^ *P* < 0.05 in comparisons of the *SiTOP1MT* group and the *SiTOP1MT* with oxamate group.

Changes in EMT markers are observed as an early step in the process of tumor metastasis. In order to evaluate changes in EMT markers in GC metastasis induced by *TOP1MT* silencing *in vivo*, we examined the expression of vimentin, fibronectin and E-cadherin, which are important EMT markers that are involved in cell migration and invasion. RT-PCR (Fig. 5A) and Western blotting (Fig. 5B) results showed that in both *shTOP1MT*/BGC-823 and SGC-7901 GC cell lines, the expression levels of vimentin and fibronectin were significantly higher, whereas E-cadherin expression was significantly lower than those in negative control cells.

Next, we examined whether LDHA was associated with changes in EMT markers induced by *TOP1MT* silencing *in vitro*. We found that *TOP1MT* silencing upregulated vimentin and fibronectin mRNA and protein and downregulated E-cadherin mRNA and protein. However, inhibition of LDHA by sodium oxamate counteracted these effects in both BGC-823 and SGC-7901 cells (Fig. 5C and D). These results indicated that changes in EMT markers may be involved in GC progression induced by upregulation of LDHA in TOP1MT-deficient cells. However, *TOP1MT* knockdown did not increase migration and invasion markers of GC cells cultured in galactose medium. Vimentin, fibronectin and E-cadherin expression had no difference between *siTOP1MT* and the control or oxamate-treated group in GC cells cultured in galactose medium (Supplementary Fig. 5A, B and C). The results further indicated that glycolysis specifically is required for increased cancer cell invasiveness.

### *TOP1MT* was linked with the clinicopathological characteristics of GC

Both *in vitro* and *in vivo* experiments demonstrated that *TOP1MT* deficiency promoted GC invasion and migration by enhancing LDHA expression. To further confirm this hypothesis in clinical specimens, we assayed tumor specimens from 295 patients with GC. Using immunohistochemical staining, we analyzed the relationships between TOP1MT/LDHA expression and clinicopathological features in patients with GC (Supplementary Tables 3 and 4).

**Figure 6:**
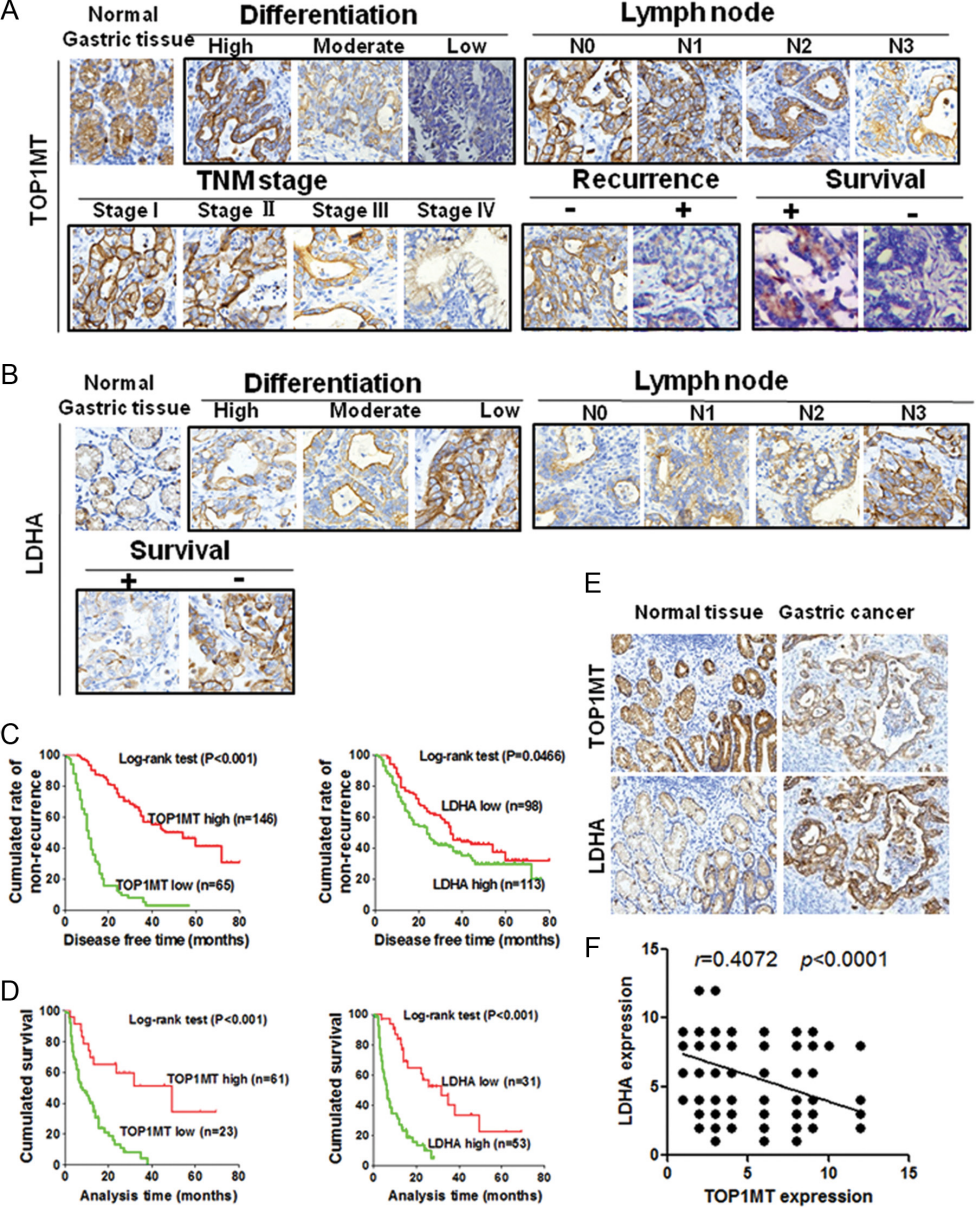
*TOP1MT* expression in patients with GC and its correlation with the clinicopathological characteristics of GC. Immunohistochemical staining of 295 primary GC specimens for *TOP1MT* or LDHA protein was performed using paraffin-embedded GC tissue specimens. (A) Representative images of immunohistochemical staining of *TOP1MT* expression in each group related to differentiation, lymph node metastasis, TNM stage, recurrence, and survival (magnification, 200×). (B) Representative images of immunohistochemical staining of LDHA expression in each group related to differentiation, lymph node metastasis, and survival (magnification, 200×). (C and D) Survival rates of patients with stage IV GC were analyzed by the Kaplan–Meier method. Log-rank tests were used to assess the survival of patients with stage IV GC in relation to *TOP1MT* (C) or LDHA (D) expression. (E) Representative images of immunohistochemical staining of *TOP1MT* and LDHA expression in consecutive sections. (F) Direct relationship between *TOP1MT* and LDHA expression in paraffin-embedded GC tissue specimens (*n* = 295; Pearson correlation coefficient, *r* = 0.4072; *P* < 0.0001). *P <0.05. A full colour version of this figure is available at http://dx.doi.org/10.1530/ERC-17-0058.

TOP1MT expression was significantly decreased in GC specimens relative to normal gastric samples by immunohistochemical staining using monoclonal anti-TOP1MT antibodies. Moreover, TOP1MT expression was significantly lower in patients with GC showing lower differentiation, more regional lymph node metastasis, more advanced stage disease and recurrence in stages I–III, and in cases of death in patients with stage IV disease during follow-up (Fig. 6A and Supplementary Fig. 6A). Immunohistochemical staining of LDHA in consecutive sections for these 295 patients with GC showed that LDHA expression was significantly higher in patients with GC having lower differentiation or more regional lymph node metastasis and in cases of death in patients with stage IV disease during follow-up (Fig. 6B and Supplementary Fig. 6B). The disease-free survival rates of patients with stage I-III cancer and the overall survival rates of patients with stage IV cancer were shorter in patients with low TOP1MT expression than those in patients with high TOP1MT expression (Fig. 6C and D). By analyzing consecutive GC sections, we found that TOP1MT expression was significantly associated with LDHA expression (Fig. 6E and F). These clinical results were consistent with the results of our *in vitro* and *in vivo* experiments using GC cell lines.

## Discussion

To the best of our knowledge, this is the first paper to study the function of *TOP1MT* in GC. We demonstrated the critical role of *TOP1MT* in promoting GC progression by regulating aerobic glycolysis. To evaluate the role of *TOP1MT* in enhancing GC invasion and migration and the underlying metabolic mechanism, we provided evidence in two GC cell lines *in vitro* and *in vivo* and clinical data for 295 patients with GC. We found that *TOP1MT* expression was lower in GC tissue specimens and cell lines than that in normal gastric tissues and cell lines. Knockdown of *TOP1MT* expression promoted the metastasis of GC cells *in vitro* and *in vivo*. *TOP1MT* deficiency significantly increased glucose consumption, lactate production, ATP production and Glut1 expression, indicating that glycolysis was enhanced. In addition, *TOP1MT* deficiency significantly increased the mRNA and protein expression levels and activity of LDHA, a key enzyme involved in glycolysis. However, LDHA inhibition counteracted GC invasion and metastasis induced by *TOP1MT* deficiency. This result suggested that the enhancement of GC invasion and metastasis induced by *TOP1MT* deficiency was associated with LDHA in GC cells. Upregulation of LDHA in *TOP1MT*-knockdown GC cells changed the EMT markers to promote cell metastasis. In order to further verify the role of *TOP1MT* in GC, we analyzed the clinical data of 295 patients with GC and found that *TOP1MT* deficiency was related to the increased tumor metastasis or recurrence and shorter survival time. Therefore, our present study results indicated that *TOP1MT* may be an underlying target gene regulating tumor metabolism, potentially contributing to the development of novel therapeutic strategies in clinical oncology.

For the first time, we assessed the expression and role of *TOP1MT* in cancer cells. To clarify its role in GC, cell cycle and cell apoptosis were analyzed by flow cytometry (data not shown), and cell proliferation was determined by MTT and colony-formation assays. Consequently, we found that *TOP1MT* deficiency did not affect GC cell cycle distribution, apoptosis (data not shown) and proliferation, consistent with previous results (Douarre *et al*. 2012). Studies have demonstrated that *TOP1MT* is critical for maintaining normal cellular metabolism in human cells; it can maintain mitochondrial physiological functions and promote OXPHOS to generate cellular ATP. *TOP1MT* deficiency leads to the dysfunction of mitochondria and enhances glycolysis in normal cells (Douarre *et al*. 2012). Since *TOP1MT* is vital for the expression of the OXPHOS-related genes in mitochondrial DNA, we hypothesize that *TOP1MT* deficiency may switch cellular glucose metabolism from OXPHOS to glycolysis because of the impaired OXPHOS in cancer cells.

Our results showed that *TOP1MT* deficiency inhibited COX expression in GC cells, indicating that *TOP1MT* deficiency was associated with decreased mitochondrial OXPHOS activity. Moreover, *TOP1MT* knockdown attenuated the expression of COX, a part of complex IV of the electron transport chain. ROS are byproducts of the mitochondrial electron transport chain (Stowe & Camara 2009). Thus, ROS production decreased because of regulation of the electron transport chain during mitochondrial respiration. Low levels of ROS suppress anoikis of tumor cells, promoting cancer metastasis (Li *et al*. 1999, Kamarajugadda *et al*. 2012).

We found that *TOP1MT* deficiency significantly increased GC cell aerobic glycolysis, also called the Warburg effect. Studies have demonstrated that the Warburg effect is the main pathway of glucose metabolism through which cancer cells generate energy (in the form of ATP) and promote biosynthesis, even under aerobic conditions (Warburg 1956, Gatenby & Gillies 2004, Semenza 2008). In fact, only 5% of glucose is metabolized by the OXPHOS pathway in mitochondria in malignant cells (Vander Heiden *et al*. 2009). *TOP1MT* deficiency may further inhibit the OXPHOS pathway of glucose metabolism and upregulate the Warburg effect in GC cells. Because cancer cells show rapid proliferation and enhanced invasiveness, oxygen consumption is increased to satisfy the multiple biosynthetic needs of cancer cells. Thus, intratumoral hypoxia is a universal characteristic of rapidly growing tumors. This hypoxic environment promotes HIF-1 activation, which activates its target genes (Kaelin & Ratcliffe 2008). *LDHA* is a transcriptional target of HIF-1. Thus, HIF-1 activation induces the expression of LDHA enzyme, which promotes pyruvate-to-lactate conversion and decreases the pyruvate flux into mitochondria. This process may stimulate glycolysis and weaken mitochondrial OXPHOS. The enhanced Warburg effect produces more lactate and thus promotes the formation of an acidic microenvironment. This may represent another mechanism through which the metastasis and invasion of tumor cells are increased (Baumann *et al*. 2009, Hirschhaeuser *et al*. 2011, Vegran *et al*. 2011).

In contrast, the upregulation of *TOP1MT* in tumor cells may downregulate the Warburg effect, shifting glucose metabolism from glycolysis to oxidative metabolism. This glucose metabolic normalization stimulates the TCA cycle and OXPHOS and increases ROS generation, resulting in inhibition of the progression of certain types of cancer cells (Bonnet *et al*. 2007). This may be a novel strategy for reversing the malignant biological behaviors of tumors. However, this hypothesis still needs to be explored further.

We confirmed that in *TOP1MT*-deficient GC cells, the enhanced Warburg effect increased tumor cell invasion and metastasis and promoted the EMT by upregulated LDHA. This result is consistent with previous findings demonstrating that the Warburg effect promotes tumor metastasis (Hsu & Sabatini 2008, Hirschhaeuser *et al*. 2011, Chen 2012, Lu *et al*. 2015). Moreover, previous studies of colorectal cancer (Koukourakis *et al*. 2006), breast cancer (Wang *et al*. 2012) and non-small cell-lung cancer (Koukourakis *et al*. 2003) have demonstrated that LDHA expression and activity are elevated in neoplasms. LDHA is one of the most important enzymes controlling the speed of glycolysis and promoting cancer progression (Le *et al*. 2010, Zhang *et al*. 2012, Augoff *et al*. 2015). However, we found that LDHA expression and activity were further increased in *TOP1MT*-deficient GC cells. LDHA inhibition significantly counteracted GC invasion and metastasis and the EMT caused by *TOP1MT* deficiency. This indicated that *TOP1MT* deficiency promoted the invasion and metastasis of tumor cells mainly through the Warburg effect and that LDHA may have played an important role in this process. Although our present study demonstrated the role of *TOP1MT* in GC metastasis and the underlying mechanisms responsible for this progression, we still plan to carry out further studies to confirm the role of *TOP1MT* in GC, including inhibiting *LDHA* by siRNA or shRNA *in vitro* and *in vivo*, which will yield more evidence to support our findings.

In summary, this report was the first to focus on the role of *TOP1MT* in tumors. We identified a new mechanism underlying *TOP1MT*-deficient GC metastasis and showed that *TOP1MT* played an important role in cancer cells. Therefore, upregulation of *TOP1MT* and application of LDHA inhibitors may inhibit GC metastasis.

## Supplementary Material

Supporting Figure 1

Supporting Figure 2

Supporting Figure 3

Supporting Figure 4

Supporting Figure 5

Supporting Figure 6

Supporting Table 1

Supporting Table 2

Supporting Table 3

Supporting Table 4

## Declaration of interest

The authors declare there is no conflict of interest that could be perceived as prejudicing the impartiality of the research reported.

## Funding

This work was supported by the National Natural Science Foundation of China (grant nos. 81502116 and 81472314) and the Special Foundation for National Clinical Specialties of China (to the Department of Oncology, Nanfang Hospital).

## References

[bib1] Augoff K Hryniewicz-Jankowska A Tabola R 2015 Lactate dehydrogenase 5: an old friend and a new hope in the war on cancer. Cancer Letters 358 1–7. (10.1016/j.canlet.2014.12.035) 25528630

[bib2] Baumann F Leukel P Doerfelt A Beier CP Dettmer K Oefner PJ Kastenberger M Kreutz M Nickl-Jockschat T Bogdahn U 2009 Lactate promotes glioma migration by TGF-beta2-dependent regulation of matrix metalloproteinase-2. Neuro-Oncology 11 368–380. (10.1215/15228517-2008-106) 19033423 PMC2743217

[bib3] Bonnet S Archer SL Allalunis-Turner J Haromy A Beaulieu C Thompson R Lee CT Lopaschuk GD Puttagunta L Bonnet S 2007 A mitochondria-K+ channel axis is suppressed in cancer and its normalization promotes apoptosis and inhibits cancer growth. Cancer Cell 11 37–51. (10.1016/j.ccr.2006.10.020) 17222789

[bib4] Chen EI 2012 Mitochondrial dysfunction and cancer metastasis. Journal of Bioenergetics and Biomembranes 44 619–622. (10.1007/s10863-012-9465-9) 22892817

[bib5] DiMauro S Schon EA 1998 Nuclear power and mitochondrial disease. Nature Genetics 19 214–215. (10.1038/883) 9662387

[bib6] Douarre C Sourbier C Dalla Rosa I Brata Das B Redon CE Zhang H Neckers L Pommier Y 2012 Mitochondrial topoisomerase I is critical for mitochondrial integrity and cellular energy metabolism. PLoS ONE 7 e41094 (10.1371/journal.pone.0041094) 22911747 PMC3401127

[bib7] Fantin VR St-Pierre J Leder P 2006 Attenuation of LDH-A expression uncovers a link between glycolysis, mitochondrial physiology, and tumor maintenance. Cancer Cell 9 425–434. (10.1016/j.ccr.2006.04.023) 16766262

[bib8] Fiume L Manerba M Vettraino M Di Stefano G 2014 Inhibition of lactate dehydrogenase activity as an approach to cancer therapy. Future Medicinal Chemistry 6 429–445. (10.4155/fmc.13.206) 24635523

[bib9] Ganapathy V Thangaraju M Prasad PD 2009 Nutrient transporters in cancer: relevance to Warburg hypothesis and beyond. Pharmacology and Therapeutics 121 29–40. (10.1016/j.pharmthera.2008.09.005) 18992769

[bib10] Gatenby RA Gillies RJ 2004 Why do cancers have high aerobic glycolysis? Nature Reviews Cancer 4 891–899. (10.1038/nrc1478) 15516961

[bib11] Giatromanolaki A Sivridis E Gatter KC Turley H Harris AL Koukourakis MI Tumour and Angiogenesis Research Group 2006 Lactate dehydrogenase 5 (LDH-5) expression in endometrial cancer relates to the activated VEGF/VEGFR2(KDR) pathway and prognosis. Gynecologic Oncology 103 912–918. (10.1016/j.ygyno.2006.05.043) 16837029

[bib12] Goto Y Hayashi R Kang D Yoshida K 2006 Acute loss of transcription factor E2F1 induces mitochondrial biogenesis in HeLa cells. Journal of Cellular Physiology 209 923–934. (10.1002/jcp.20802) 16972274

[bib13] Hirschhaeuser F Sattler UG Mueller-Klieser W 2011 Lactate: a metabolic key player in cancer. Cancer Research 71 6921–6925. (10.1158/0008-5472.CAN-11-1457) 22084445

[bib14] Hsu PP Sabatini DM 2008 Cancer cell metabolism: Warburg and beyond. Cell 134 703–707. (10.1016/j.cell.2008.08.021) 18775299

[bib15] Kaelin WG Jr Ratcliffe PJ 2008 Oxygen sensing by metazoans: the central role of the HIF hydroxylase pathway. Molecular Cell 30 393–402. (10.1016/j.molcel.2008.04.009) 18498744

[bib16] Kamarajugadda S Stemboroski L Cai Q Simpson NE Nayak S Tan M Lu J 2012 Glucose oxidation modulates anoikis and tumor metastasis. Molecular and Cellular Biology 32 1893–1907. (10.1128/MCB.06248-11) 22431524 PMC3347404

[bib17] Kolev Y Uetake H Takagi Y Sugihara K 2008 Lactate dehydrogenase-5 (LDH-5) expression in human gastric cancer: association with hypoxia-inducible factor (HIF-1alpha) pathway, angiogenic factors production and poor prognosis. Annals of Surgical Oncology 15 2336–2344. (10.1245/s10434-008-9955-5) 18521687

[bib18] Koukourakis MI Giatromanolaki A Sivridis E Bougioukas G Didilis V Gatter KC Harris AL Tumour and Angiogenesis Research Group 2003 Lactate dehydrogenase-5 (LDH-5) overexpression in non-small-cell lung cancer tissues is linked to tumour hypoxia, angiogenic factor production and poor prognosis. British Journal of Cancer 89 877–885. (10.1038/sj.bjc.6601205) 12942121 PMC2394471

[bib19] Koukourakis MI Giatromanolaki A Sivridis E Gatter KC Harris AL Tumour and Angiogenesis Research Group 2006 Lactate dehydrogenase 5 expression in operable colorectal cancer: strong association with survival and activated vascular endothelial growth factor pathway—a report of the Tumour Angiogenesis Research Group. Journal of Clinical Oncology 24 4301–4308. (10.1200/JCO.2006.05.9501) 16896001

[bib20] Koukourakis MI Giatromanolaki A Winter S Leek R Sivridis E Harris AL 2009 Lactate dehydrogenase 5 expression in squamous cell head and neck cancer relates to prognosis following radical or postoperative radiotherapy. Oncology 77 285–292. (10.1159/000259260) 19923867

[bib21] Kroemer G Pouyssegur J 2008 Tumor cell metabolism: cancer’s Achilles’ heel. Cancer Cell 13 472–482. (10.1016/j.ccr.2008.05.005) 18538731

[bib22] Le A Cooper CR Gouw AM Dinavahi R Maitra A Deck LM Royer RE Vander Jagt DL Semenza GL Dang CV 2010 Inhibition of lactate dehydrogenase A induces oxidative stress and inhibits tumor progression. PNAS 107 2037–2042. (10.1073/pnas.0914433107) 20133848 PMC2836706

[bib23] Li AE Ito H Rovira II Kim KS Takeda K Yu ZY Ferrans VJ Finkel T 1999 A role for reactive oxygen species in endothelial cell anoikis. Circulation Research 85 304–310. (10.1161/01.RES.85.4.304) 10455058

[bib24] Lin L Huang H Liao W Ma H Liu J Wang L Huang N Liao Y Liao W 2015 MACC1 supports human gastric cancer growth under metabolic stress by enhancing the Warburg effect. Oncogene 34 2700–2710. (10.1038/onc.2014.204) 25043301

[bib25] Lu J Tan M Cai Q 2015 The Warburg effect in tumor progression: mitochondrial oxidative metabolism as an anti-metastasis mechanism. Cancer Letters 356 156–164. (10.1016/j.canlet.2014.04.001) 24732809 PMC4195816

[bib26] Semenza GL 2008 Tumor metabolism: cancer cells give and take lactate. Journal of Clinical Investigation 118 3835–3837. (10.1172/JCI37373) 19033652 PMC2582934

[bib27] Sotgia F Whitaker-Menezes D Martinez-Outschoorn UE Flomenberg N Birbe RC Witkiewicz AK Howell A Philp NJ Pestell RG Lisanti MP 2012 Mitochondrial metabolism in cancer metastasis: visualizing tumor cell mitochondria and the ‘reverse Warburg effect’ in positive lymph node tissue. Cell Cycle 11 1445–1454. (10.4161/cc.19841) 22395432 PMC3350881

[bib28] Stowe DF Camara AK 2009 Mitochondrial reactive oxygen species production in excitable cells: modulators of mitochondrial and cell function. Antioxidants and Redox Signaling 11 1373–1414. (10.1089/ars.2008.2331) 19187004 PMC2842133

[bib29] Vander Heiden MG Cantley LC Thompson CB 2009 Understanding the Warburg effect: the metabolic requirements of cell proliferation. Science 324 1029–1033. (10.1126/science.1160809) 19460998 PMC2849637

[bib30] Vegran F Boidot R Michiels C Sonveaux P Feron O 2011 Lactate influx through the endothelial cell monocarboxylate transporter MCT1 supports an NF-kappaB/IL-8 pathway that drives tumor angiogenesis. Cancer Research 71 2550–2560. (10.1158/0008-5472.CAN-10-2828) 21300765

[bib32] Wang ZY Loo TY Shen JG Wang N Wang DM Yang DP Mo SL Guan XY Chen JP 2012 LDH-A silencing suppresses breast cancer tumorigenicity through induction of oxidative stress mediated mitochondrial pathway apoptosis. Breast Cancer Research and Treatment 131 791–800. (10.1007/s10549-011-1466-6) 21452021

[bib31] Wang L Wu Y Lin L Liu P Huang H Liao W Zheng D Zuo Q Sun L Huang N 2013 Metastasis-associated in colon cancer-1 upregulation predicts a poor prognosis of gastric cancer, and promotes tumor cell proliferation and invasion. International Journal of Cancer 133 1419–1430. (10.1002/ijc.28140) 23457029

[bib33] Warburg O 1956 On the origin of cancer cells. Science 123 309–314. (10.1126/science.123.3191.309) 13298683

[bib34] Xia J Wang H Huang H Sun L Dong S Huang N Shi M Bin J Liao Y Liao W 2016 Elevated orai1 and STIM1 expressions upregulate MACC1 expression to promote tumor cell proliferation, metabolism, migration, and invasion in human gastric cancer. Cancer Letters 381 31–40. (10.1073/pnas.191321998) 27431311

[bib35] Zhang H Barcelo JM Lee B Kohlhagen G Zimonjic DB Popescu NC Pommier Y 2001 Human mitochondrial topoisomerase I. PNAS 98 10608–10613. (10.1073/pnas.191321998) 11526219 PMC58513

[bib36] Zhang Y Zhang X Wang X Gan L Yu G Chen Y Liu K Li P Pan J Wang J 2012 Inhibition of LDH-A by lentivirus-mediated small interfering RNA suppresses intestinal-type gastric cancer tumorigenicity through the downregulation of Oct4. Cancer Letters 321 45–54. (10.1016/j.canlet.2012.03.013) 22429998

