# Supplementary material for: TOP1MT deficiency promotes GC invasion and migration via the enhancements of LDHA expression and aerobic glycolysis
Source: Endocr Relat Cancer. 2017 Sep 5;24(11):565–78. doi: 10.1530/ERC-17-0058 (PMC5633043; doi:10.1530/ERC-17-0058)

Supplemental figure 1

SGC-7901 (passage number: 13)

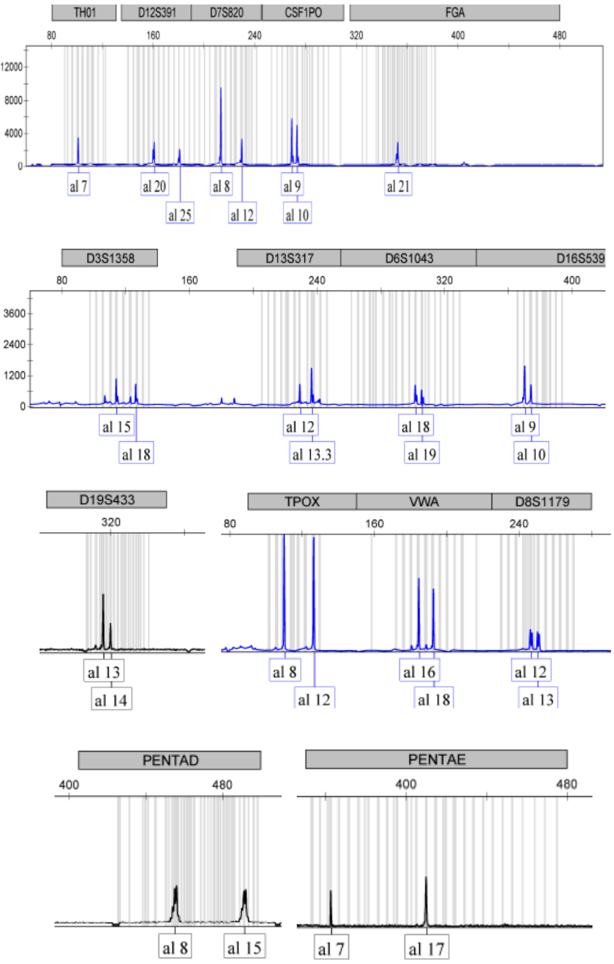

MKN-7 (passage number: 14)

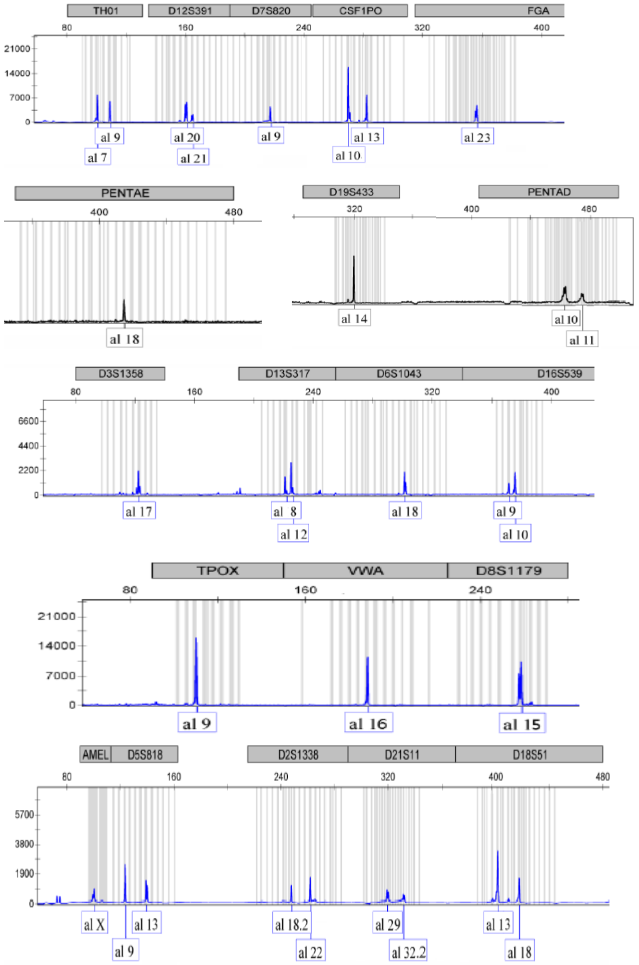

MKN-45 (passage number: 11)

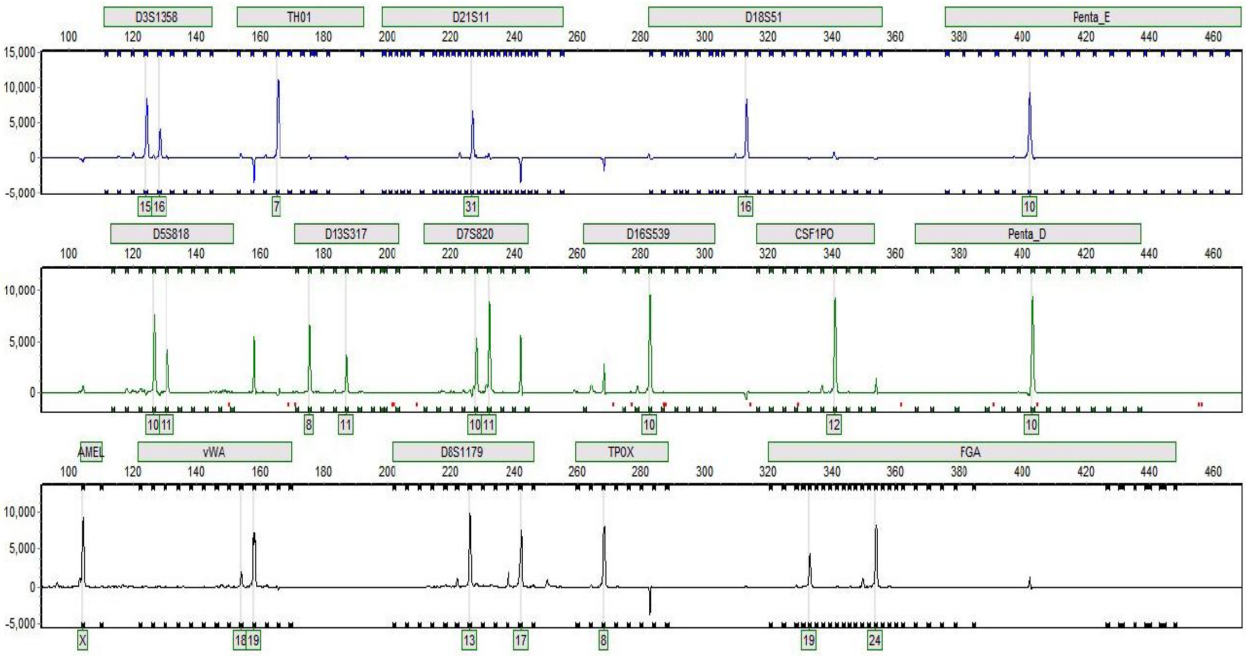

AGS (passage number: 14)

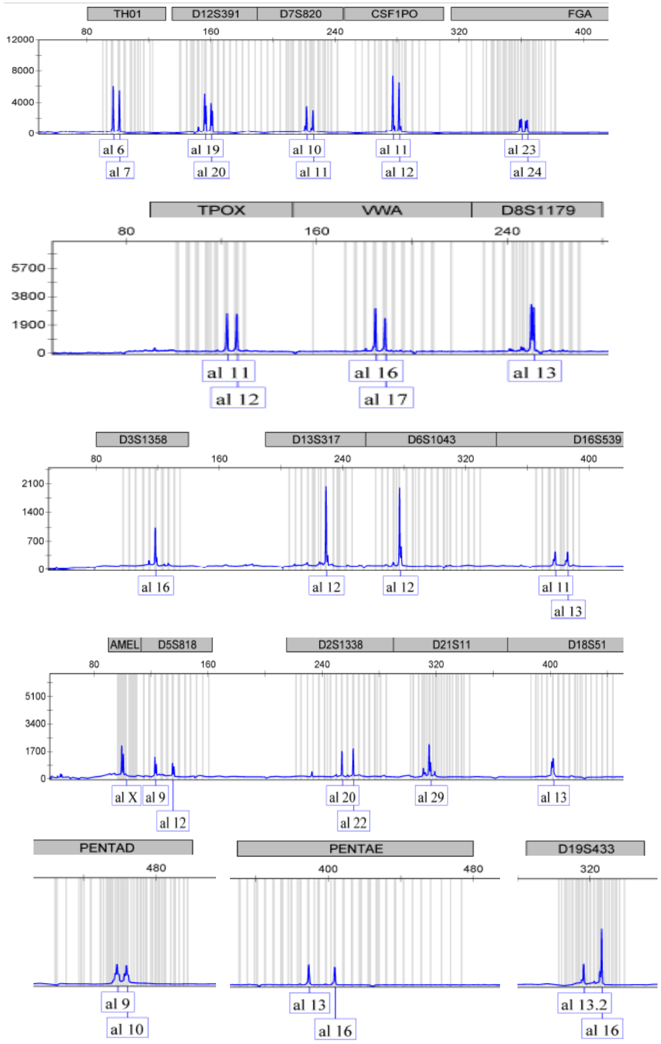

MGC-803 (passage number: 13)

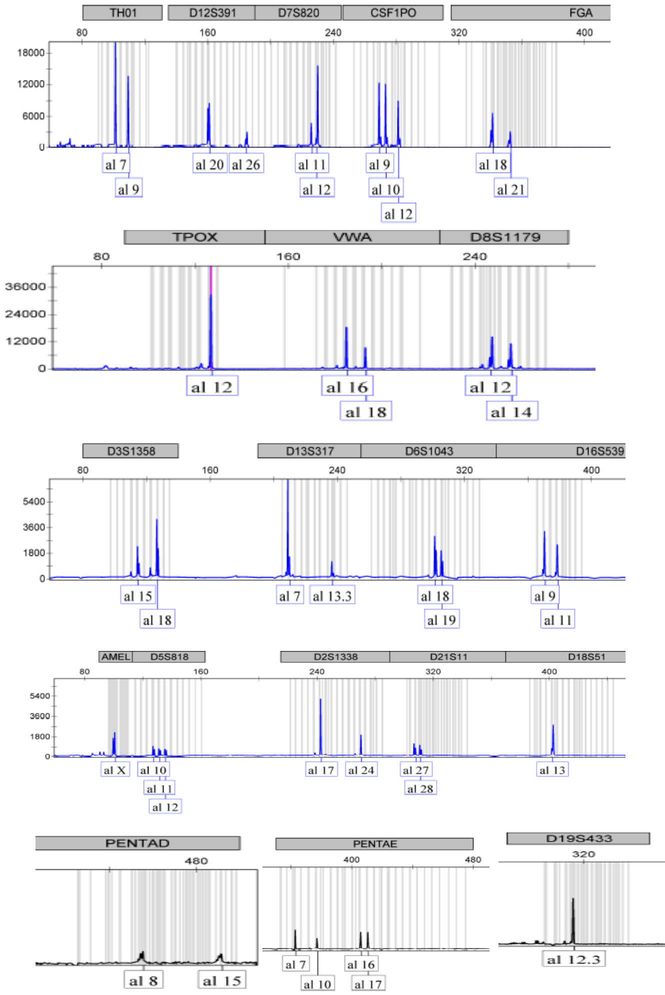

MKN-28 (passage number: 14)

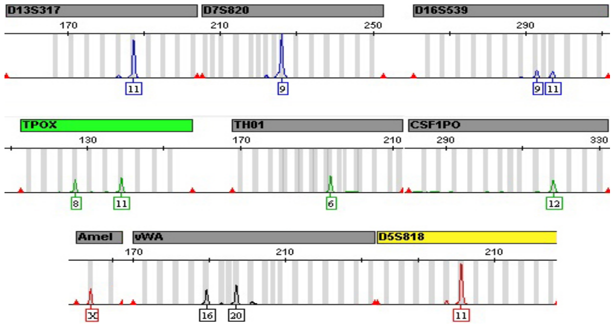

BGC-823 (passage number: 11)

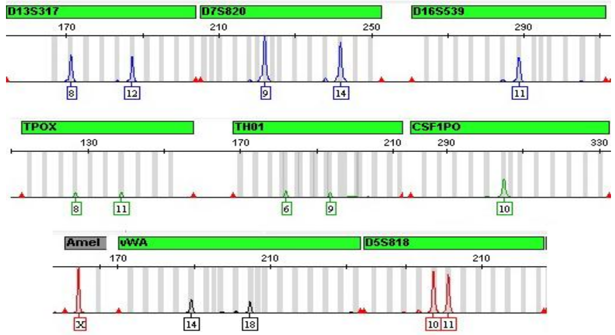

GES-1 (passage number: 15)

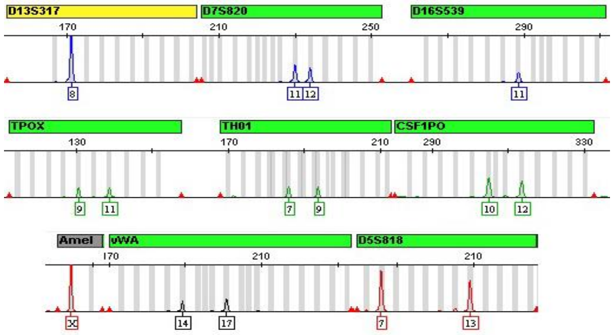

Supplement: Supporting Figure 1 [file erc-24-565-s001.pdf]
