# Supplementary figures and images for: TOP1MT deficiency promotes GC invasion and migration via the enhancements of LDHA expression and aerobic glycolysis
Source: Endocr Relat Cancer. 2017 Sep 5;24(11):565–78. doi: 10.1530/ERC-17-0058 (PMC5633043; doi:10.1530/ERC-17-0058)

## Supplemental figure 2

A

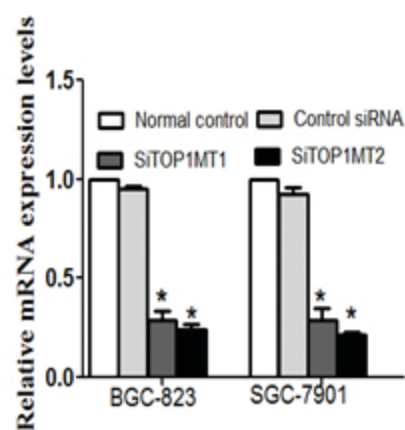

B

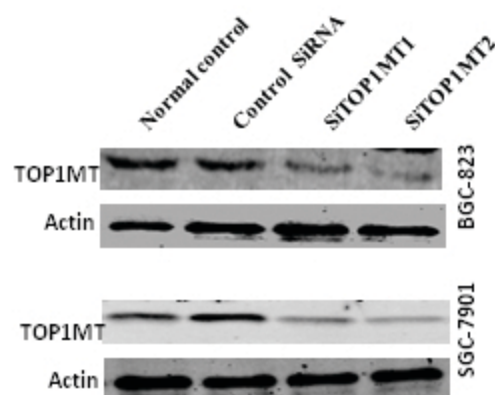

C

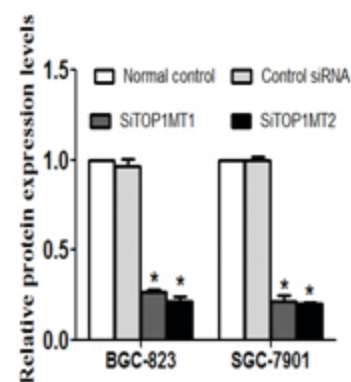

Supplement: Supporting Figure 2 [file erc-24-565-s002.pdf]

# Supplemental figure 3

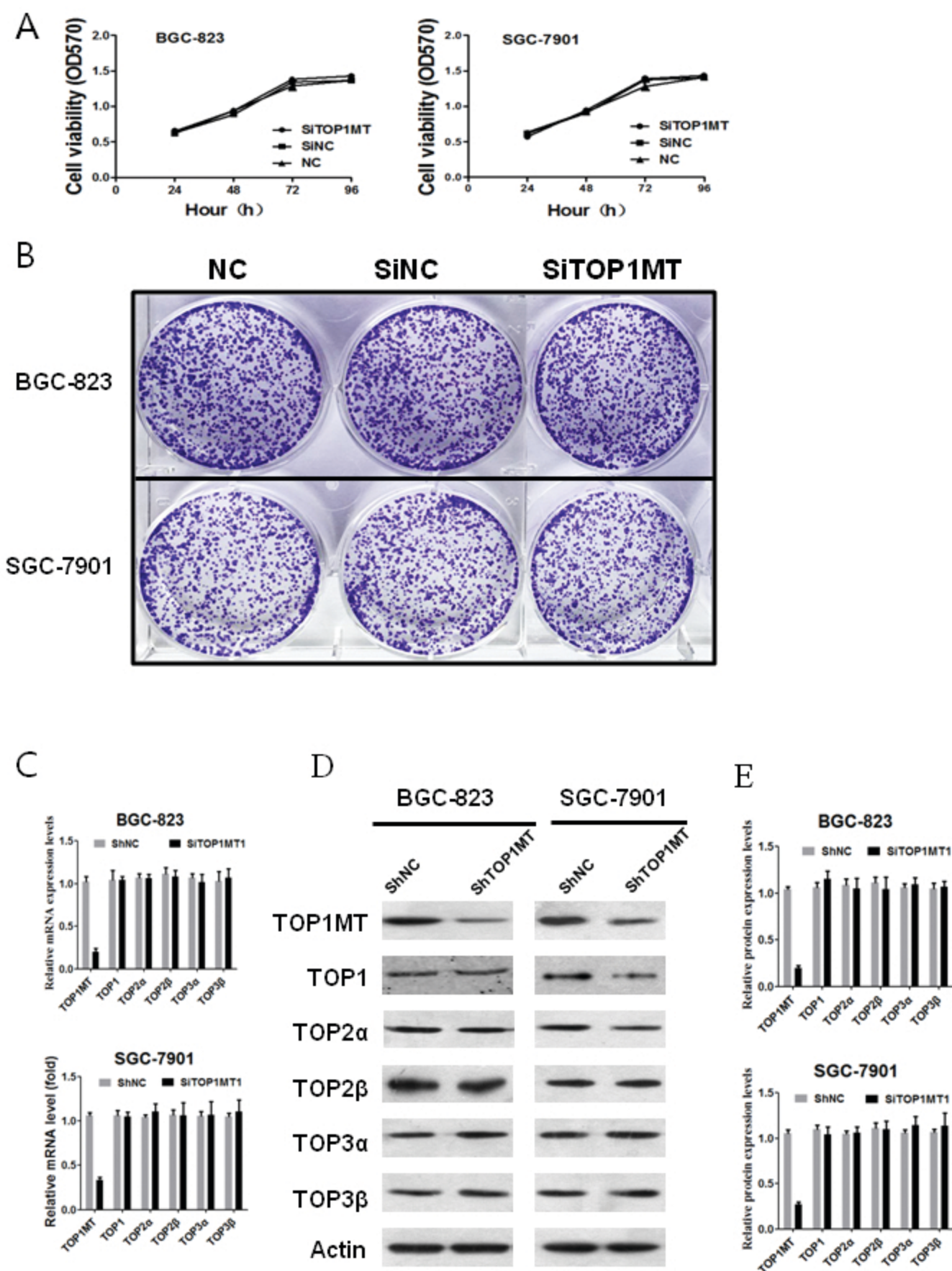

Supplement: Supporting Figure 3 [file erc-24-565-s003.pdf]

# Supplemental figure 4

A

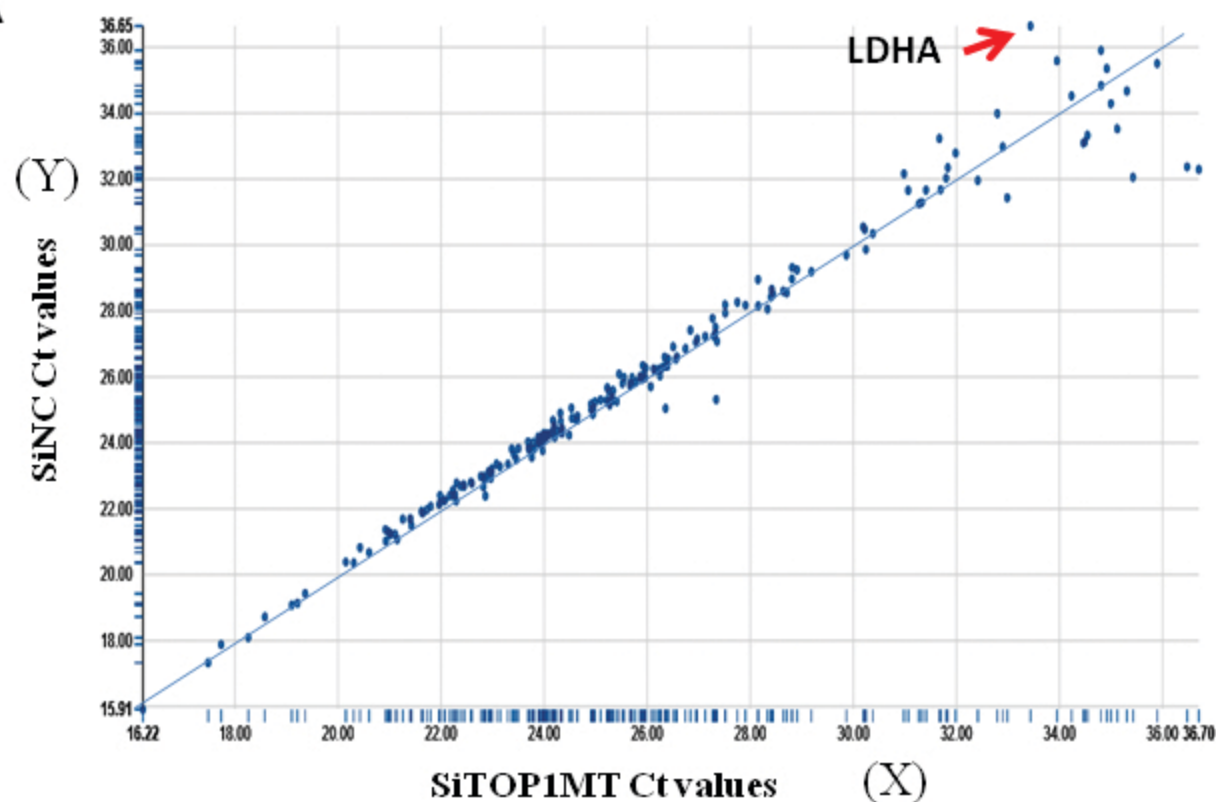

B

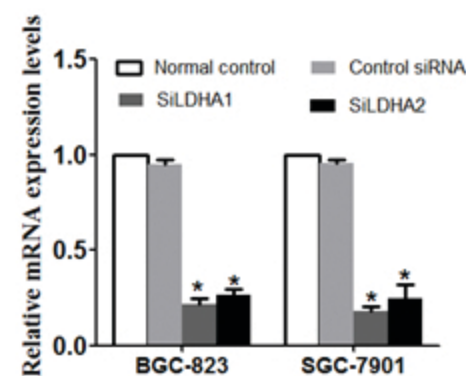

C

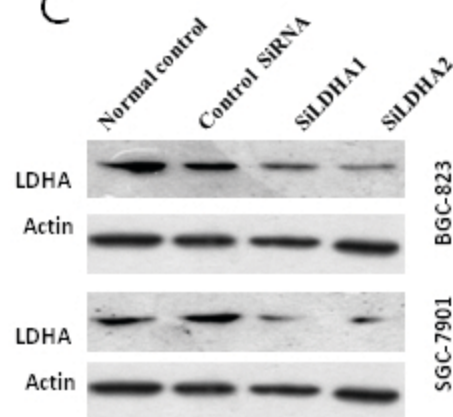

D

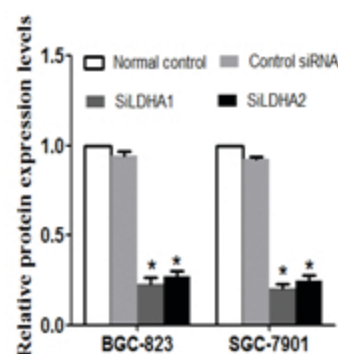

E

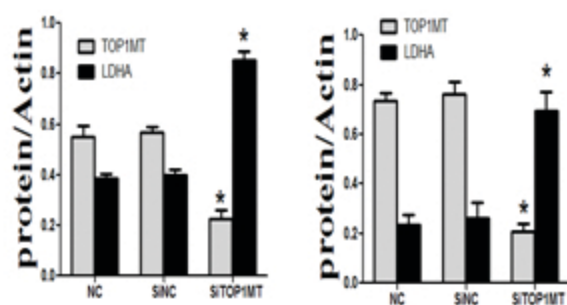

F

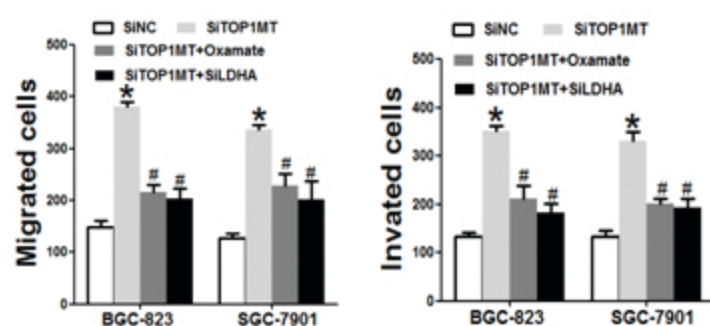

Supplement: Supporting Figure 4 [file erc-24-565-s004.pdf]

# Supplemental figure 5

A

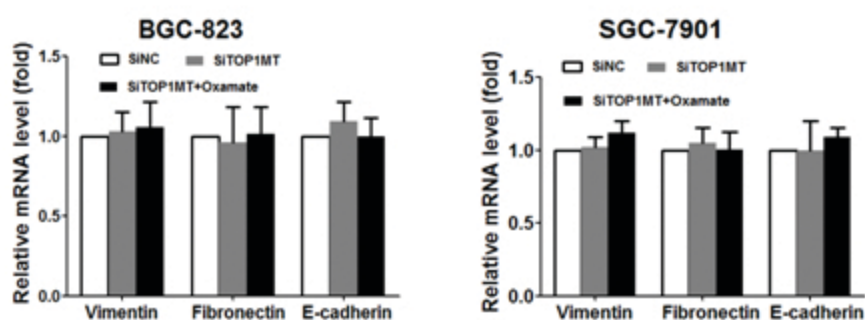

B

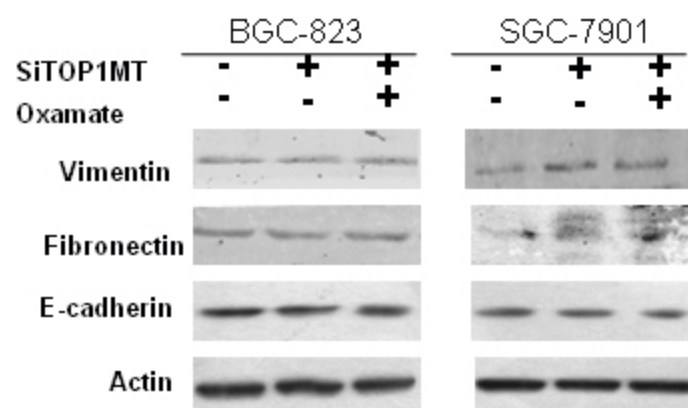

C

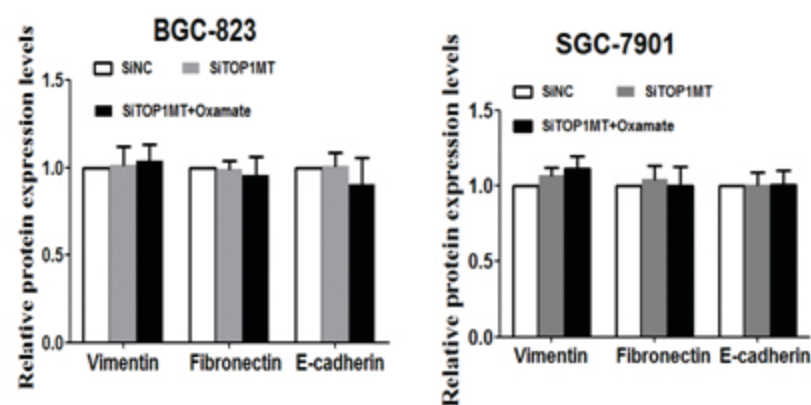

Supplement: Supporting Figure 5 [file erc-24-565-s005.pdf]

## Supplemental figure 6

A

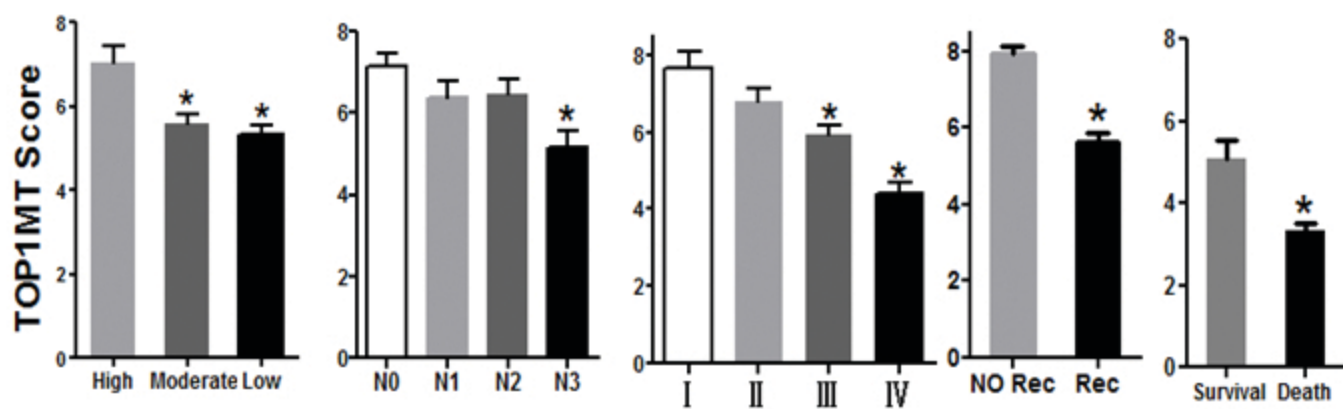

B

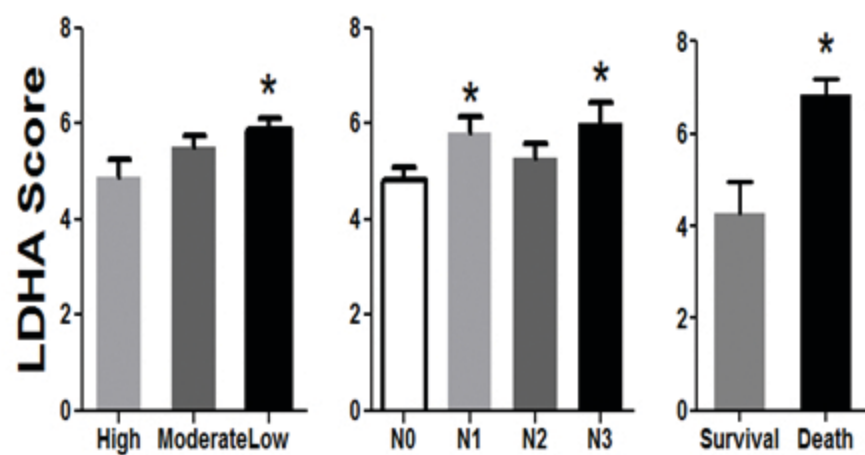

Supplement: Supporting Figure 6 [file erc-24-565-s006.pdf]
