# Supplementary material for: TOP1MT deficiency promotes GC invasion and migration via the enhancements of LDHA expression and aerobic glycolysis
Source: Endocr Relat Cancer. 2017 Sep 5;24(11):565–78. doi: 10.1530/ERC-17-0058 (PMC5633043; doi:10.1530/ERC-17-0058)
Supplement: Supporting Table 1 [file erc-24-565-t001.pdf]

Supplemental Table 1. Primers used for reverse transcription (RT)-PCR analyses

Gene name	Primer sequence (5` to 3`)	Primer length (bp)
TOP1MT	FW: CGGTGGCCCTGTATTTCAT	19
	RV: GCCTCACCGTCCTCCCTC	18
LDHA	FW: GTTCACAAGCAGGT	14
	RV: GGTTGAGAGTGCTT	14
Vimentin	FW: CGCCAGATGCGTGAAATGG	19
	RV: ACCAGAGGGAGTGAATCCAGA	21
Fibronectin	FW: ATGATGAGGTGCACGTGTGT	20
	RV: CCCTGACCGAAGCATGTACA	20
E-cadherin	FW: ATGAGTGTCCCCCGGTATCT	20
	RV: CAAACACGAGCAGAGAATCA	20
Glut1	FW: TACCCTGGATGTCCTATCTG	20
	RV: CACACAGTTGCTCCACATAC	20
β-***actin***	FW: TGACGGGGTCACCCACACTGTGCCCATCTA	30
	RV: GAAGCATTTGCGGTGGACGA TGGAGGG	27

TOP1MT: mtochondrial topoisomerase I; ***LDHA:*** lactate dehydrogenase A; ***Glut1:*** glucose transporter 1
