# Supplementary material for: TOP1MT deficiency promotes GC invasion and migration via the enhancements of LDHA expression and aerobic glycolysis
Source: Endocr Relat Cancer. 2017 Sep 5;24(11):565–78. doi: 10.1530/ERC-17-0058 (PMC5633043; doi:10.1530/ERC-17-0058)
Supplement: Supporting Table 2 [file erc-24-565-t002.pdf]

Supplemental Table 2. Short interfering RNA sequence (siTOP1MT1 and siTOP1MT2) targeting TOP1MT

siTOP1MT	sequence(5` to 3`)
**siTOP1MT1**	CCAACACGUGGUGGAAUUUTT
**siTOP1MT2**	GCAUAGCAGCUAAGAUCUUTT
**siLDHA1**	GGACTTGGCAGATGAACTT
**siLDHA2**	CC TTAGAACACCAAAGATT
