# Supplementary material for: TOP1MT deficiency promotes GC invasion and migration via the enhancements of LDHA expression and aerobic glycolysis
Source: Endocr Relat Cancer. 2017 Sep 5;24(11):565–78. doi: 10.1530/ERC-17-0058 (PMC5633043; doi:10.1530/ERC-17-0058)
Supplement: Supporting Table 3 [file erc-24-565-t003.pdf]

Supplemental Table 3. Clinical pathological characteristics of gastric cancer patients with high and low TOP1MT immunohistochemical staining

Characteristic	*n* (%)	TOP1MT expression		*p*-value
		Low (*n*, %)	High (*n*, %)	
**Age (year)**				0.516
≥55	200 (67.8%)	88 (44.0%)	112 (56.0%)	
<55	95 (32.2%)	38 (40.0%)	57 (60.0%)	
**Gender**				0.203
Male	201 (68.1%)	81 (40.3%)	120 (59.7%)	
Female	94 (31.9%)	45 (47.9%)	49 (52.1%)	
**Differentiation**				**0.018**
High	48 (16.2%)	12 (25.0%)	36 (75.0%)	
Moderate	113 (38.3%)	49 (43.4%)	64 (56.6%)	
Low	134 (45.4%)	65 (48.5%)	69 (51.5%)	
**Lymph note**				**0.003**
N0	73 (34.6%)	13 (17.8%)	60 (82.2%)	
N1	56 (26.5%)	16 (28.6%)	40 (71.4%)	
N2	49 (23.2%)	19 (38.8%)	30 (61.2%)	
N3	33 (15.6%)	17 (51.5%)	16 (48.5%)	
**TNM stage**				**0.000**
Ⅰ	34 (11.5%)	3 (8.8%)	31 (91.2%)	
Ⅱ	67 (22.7%)	16 (23.9%)	51 (76.1%)	
Ⅲ	110 (37.3%)	46 (41.8%)	64 58.2(%)	
Ⅳ	84 (28.5%)	61 (72.6%)	23 (27.4%)	
**Recurrence (stage I–III)**				**0.000**
Yes	133 (63.0%)	62 (46.6%)	71 (53.4%)	
No	78 (37.0%)	3 (3.8%)	75 (96.2%)	
**Survival (stage IV)**				**0.001**
Yes	21 (25.0%)	9 (42.9%)	12 (57.1%)	
No	63 (75.0%)	52 (82.5%)	11 (17.5%)	
