# Supplementary material for: TOP1MT deficiency promotes GC invasion and migration via the enhancements of LDHA expression and aerobic glycolysis
Source: Endocr Relat Cancer. 2017 Sep 5;24(11):565–78. doi: 10.1530/ERC-17-0058 (PMC5633043; doi:10.1530/ERC-17-0058)
Supplement: Supporting Table 4 [file erc-24-565-t004.pdf]

Supplemental Table 4. Clinical pathological characteristics of gastric cancer patients exhibiting high and low LDHA immunohistochemical staining

**Characteristic**	***n* (%)**	**LDHA expression**		***p*-value**
		**Low (*n*, %)**	**High (*n*, %)**	
**Age (year)**				0.633
≥55	129 (43.7%)	86 (66.7%)	43 (33.3%)	
<55	166 (56.3%)	115 (69.3%)	51 (30.7%)	
**Gender**				0.466
Male	201 (68.1%)	85 (42.3%)	116 (57.7%)	
Female	94 (31.9%)	44 (46.8%)	50 (53.2%)	
**Differentiation**				0.052
High	48 (16.3%)	28 (58.3%)	20 (41.7%)	
Moderate	113 (38.3%)	50 (44.2%)	63 (55.8%)	
Low	134 (45.4%)	51 (38.1%)	83 (61.9%)	
**Lymph note**				0.118
N0	74 (35.1%)	42 (56.8%)	32 (43.2%)	
N1	45 (21.3%)	17 (37.8%)	28 (62.2%)	
N2	59 (28.0%)	27 (45.8%)	32 (54.2%)	
N3	33 (15.6%)	12 (36.4%)	21 (63.6%)	
**TNM stage**				0.165
Ⅰ	34 (11.5%)	18 (52.9%)	16 (47.1%)	
Ⅱ	67 (22.7%)	35 (52.2%)	32 (47.8%)	
Ⅲ	110 (37.3%)	45 (40.9%)	65 (59.1%)	
Ⅳ	84 (28.5%)	31 (36.9%)	53 (63.1%)	
**Recurrence (stage I–III)**				0.623
Yes	83 (39.3%)	54 (65.1%)	29 (34.9%)	
No	128 (60.7%)	79 (61.7%)	49 (38.3%)	
**Survival (stage IV)**				**0.000**
Yes	21 (25.0%)	14 (66.7%)	7 (33.3%)	
No	63 (75.0%)	17 (27.0%)	46 (73.0%)	
